# The retrospective analysis of Antarctic tracking data project

**DOI:** 10.1038/s41597-020-0406-x

**Published:** 2020-03-18

**Authors:** Yan Ropert-Coudert, Anton P. Van de Putte, Ryan R. Reisinger, Horst Bornemann, Jean-Benoît Charrassin, Daniel P. Costa, Bruno Danis, Luis A. Hückstädt, Ian D. Jonsen, Mary-Anne Lea, David Thompson, Leigh G. Torres, Philip N. Trathan, Simon Wotherspoon, David G. Ainley, Rachael Alderman, Virginia Andrews-Goff, Ben Arthur, Grant Ballard, John Bengtson, Marthán N. Bester, Arnoldus Schytte Blix, Lars Boehme, Charles-André Bost, Peter Boveng, Jaimie Cleeland, Rochelle Constantine, Robert J. M. Crawford, Luciano Dalla Rosa, P. J. Nico de Bruyn, Karine Delord, Sébastien Descamps, Mike Double, Louise Emmerson, Mike Fedak, Ari Friedlaender, Nick Gales, Mike Goebel, Kimberly T. Goetz, Christophe Guinet, Simon D. Goldsworthy, Rob Harcourt, Jefferson T. Hinke, Kerstin Jerosch, Akiko Kato, Knowles R. Kerry, Roger Kirkwood, Gerald L. Kooyman, Kit M. Kovacs, Kieran Lawton, Andrew D. Lowther, Christian Lydersen, Phil O’B. Lyver, Azwianewi B. Makhado, Maria E. I. Márquez, Birgitte I. McDonald, Clive R. McMahon, Monica Muelbert, Dominik Nachtsheim, Keith W. Nicholls, Erling S. Nordøy, Silvia Olmastroni, Richard A. Phillips, Pierre Pistorius, Joachim Plötz, Klemens Pütz, Norman Ratcliffe, Peter G. Ryan, Mercedes Santos, Colin Southwell, Iain Staniland, Akinori Takahashi, Arnaud Tarroux, Wayne Trivelpiece, Ewan Wakefield, Henri Weimerskirch, Barbara Wienecke, José C. Xavier, Ben Raymond, Mark A. Hindell

**Affiliations:** 1 Centre d’Etudes Biologiques de Chizé, Station d’Écologie de Chizé- La Rochelle Université, CNRS UMR7372, 79360 Villiers-en-Bois, France; 2 BEDIC, OD Nature, Royal Belgian Institute for Natural Sciences, Vautierstraat 29, B-1000 Brussels, Belgium; 3 Laboratory of Biodiversity and Evolutionary Genomics, Department of Biology, University of Leuven, Ch. Deberiotstraat 32, B-3000 Leuven, Belgium; 4 DST-NRF Centre of Excellence at the Percy FitzPatrick Institute of African Ornithology, Nelson Mandela University, PO Box 77000, Port Elizabeth, 6031 South Africa; 5 CESAB – FRB, 5, rue de l’École de médecine, 34000 Montpellier, France; 6 Alfred-Wegener-Institut Helmholtz-Zentrum für Polar- und Meeresforschung, Am Handelshafen 12, 27570 Bremerhaven, Germany; 7 Sorbonne Universités, UPMC University, Paris 06, UMR 7159 CNRS-IRD-MNHN, LOCEAN-IPSL, 75005 Paris, France; 8 Department of Ecology and Evolutionary Biology, University of California Santa Cruz, Long Marine Lab, 130 McAllister Way, Santa Cruz, CA 95060 USA; 9 Université Libre de Bruxelles, Marine Biology Lab, Campus du Solbosch - CP160/15 50 avenue F.D. Roosevelt, 1050 Bruxelles, Belgium; 10 Department of Biological Sciences, Macquarie University, Sydney, NSW 2109 Australia; 11 Institute for Marine and Antarctic Studies, University of Tasmania, 20 Castray Esplanade, Battery Point, TAS 7004 Australia; 12 Antarctic Climate and Ecosystems Cooperative Research Centre, University of Tasmania, TAS 7004, Hobart, Australia; 13 National Institute of Water and Atmospheric Research Ltd, 301 Evans Bay Parade, Wellington, 6021 New Zealand; 14 Hatfield Marine Science Center, 2030 SE Marine Science Drive, Newport, OR 97365 USA; 15 British Antarctic Survey, Natural Environment Research Council, High Cross, Madingley Road, Cambridge, CB3 0ET United Kingdom; 16 H.T. Harvey & Associates, 983 University Avenue, Bldg D, Los Gatos, CA 95032 USA; 17 Department of Primary Industries, Parks, Water and Environment, Hobart, TAS 7000 Australia; 18 Australian Antarctic Division, Department of the Environment and Energy, 203 Channel Hwy, Kingston, TAS 7050 Australia; 19 Point Blue Conservation Science, 3820 Cypress Drive, Suite 11, Petaluma, CA 94954 USA; 20 Marine Mammal Laboratory, Alaska Fisheries Science Center/NOAA, 7600 Sand Point Way N.E., F/AKC3, Seattle, WA 98115-6349 USA; 21 Mammal Research Institute, Department of Zoology and Entomology, University of Pretoria, Private Bag X20, Hatfield, 0028 South Africa; 22 UiT The Arctic University of Norway, PO Box 6050 Langnes, 9037 Tromsø, Norway; 23 Scottish Oceans Institute, East Sands, St Andrews, Fife, United Kingdom; 24 School of Biological Sciences, University of Auckland Private Bag 92019, Auckland, New Zealand; 25 Oceans and Coasts, Department of Environmental Affairs, Private Bag X2, Rogge Bay, 8012 South Africa; 26 Instituto de Oceanografia, Universidade Federal do Rio Grande - FURG, Av. Itália km 8 s/n, Campus Carreiros, Rio Grande, RS 96203-000 Brazil; 27 Norwegian Polar Institute, Fram Centre, 9296 Tromsø, Norway; 28 Institute of Marine Sciences, University of California Santa Cruz, 1156 High Street, Santa Cruz, CA 95064 USA; 29 Antarctic Ecosystem Research Division, Southwest Fisheries Science Center, National Marine Fisheries, Service, National Oceanic and Atmospheric Administration, La Jolla, California USA; 30 South Australian Research and Development Institute, 2 Hamra Avenue, West Beach, SA 5024 Australia; 31 Center for Marine Biology & Biomedicine, Scripps Institution of Oceanography, UC San Diego, La Jolla, CA 92093 USA; 32 Landcare Research, Lincoln, P.O. Box 69040, Lincoln, 7640 New Zealand; 33 Instituto Antártico Argentino, 25 de Mayo, 1143 San Martín, Provincia de Buenos Aires Argentina; 34 Moss Landing Marine Laboratories, San José State University, 8272 Moss Landing Rd, Moss Landing, CA 95039 USA; 35 Sydney Institute of Marine Science, 19 Chowder Bay Road, Mosman, NSW 2088 Australia; 36 Institute for Terrestrial and Aquatic Wildlife Research, University of Veterinary Medicine Hannover, Werftstraße 6, 25761 Büsum, Germany; 37 Dipartimento di Scienze Fisiche, della Terra e dell’Ambiente, Università di Siena, Via Mattioli 4, 53100 Siena, Italy; 38 Museo Nazionale dell’Antartide, Via Laterina 8, 53100 Siena, Italy; 39 Antarctic Research Trust, Am Oste-Hamme-Kanal 10, D-27432 Bremervörde, Germany; 40 Percy FitzPatrick Institute of African Ornithology, DST-NRF Centre of Excellence, University of Cape Town, Rondebosch, 7701 South Africa; 41 National Institute of Polar Research, 10-3, Midori-cho, Tachikawa, Tokyo, 190-8518 Japan; 42 Norwegian Institute for Nature Research, Fram Centre, Postbox 6606 Langnes, 9296 Tromsø, Norway; 43 Institute of Biodiversity Animal Health and Comparative Medicine, University of Glasgow, Glasgow, G12 8QQ United Kingdom; 44 Marine and Environmental Sciences Centre, Department of Life Sciences, University of Coimbra, 3004-517 Coimbra, Portugal

**Keywords:** Ecosystem ecology, Conservation biology

## Abstract

The Retrospective Analysis of Antarctic Tracking Data (RAATD) is a Scientific Committee for Antarctic Research project led jointly by the Expert Groups on Birds and Marine Mammals and Antarctic Biodiversity Informatics, and endorsed by the Commission for the Conservation of Antarctic Marine Living Resources. RAATD consolidated tracking data for multiple species of Antarctic meso- and top-predators to identify Areas of Ecological Significance. These datasets and accompanying syntheses provide a greater understanding of fundamental ecosystem processes in the Southern Ocean, support modelling of predator distributions under future climate scenarios and create inputs that can be incorporated into decision making processes by management authorities. In this data paper, we present the compiled tracking data from research groups that have worked in the Antarctic since the 1990s. The data are publicly available through biodiversity.aq and the Ocean Biogeographic Information System. The archive includes tracking data from over 70 contributors across 12 national Antarctic programs, and includes data from 17 predator species, 4060 individual animals, and over 2.9 million observed locations.

## Abstract (summary)

**Table:** 

Measurement(s)	geographic location
Technology Type(s)	digital curation
Factor Type(s)	temporal interval • geographic location
Sample Characteristic – Organism	Aptenodytes forsteri • Lobodon carcinophaga • Eudyptes chrysolophus • Thalassarche melanophrys • Phoebetria palpebrata • Aptenodytes patagonicus • Pygoscelis adeliae • Leptonychotes weddellii • Megaptera novaeangliae • Mirounga leonina • Arctocephalus gazella • Thalassoica antarctica • Eudyptes schlegeli • Diomedea exulans • Phoebetria fusca • Diomedea chrysostoma • Procellaria aequinoctialis
Sample Characteristic – Environment	polar biome
Sample Characteristic – Location	Antarctica

Machine-accessible metadata file describing the reported data: 10.6084/m9.figshare.11688876

## Background & Summary

There is increasing evidence and concern that Southern Ocean ecosystems are facing globally significant challenges, especially in regions undergoing some of the fastest rates of warming on Earth, or where commercial fishing may be impacting ecosystem processes. At lower latitude locations, in the west, such as the Antarctic Peninsula, winter air temperatures have warmed by 4.8 times the global average, and ocean surface temperatures have risen by 1 °C^1,2^. At the same time, concerns about commercial catches of Antarctic krill *Euphausia superba* and toothfish *Dissostichus* spp. continue, e.g.^3^. Ecological effects arising at multiple scales from the physical changes in the environment require further investigations^4–6^ to allow a realistic assessment of the effects of regional and global warming and ocean acidification vs. top predator recoveries and/or fishing^7^. The paucity of data on spatial and temporal ecosystem dynamics, and heterogeneity of change even at relatively small spatial scales, e.g.^8^, adds considerable uncertainty around projections for biological systems. Local mitigation or management measures require a solid knowledge foundation to encapsulate critical ecosystem processes or vulnerable ecosystem components^9^.

The distributions and abundances of marine endotherms in the Southern Ocean are linked to both habitat and prey availability^10^. Areas with high concentrations of predators often signal higher diversity or abundance of lower trophic organisms, and are therefore regions that may need special management consideration. In addition to a long history of at-sea surveys, e.g.^11^, recent advances in electronic tagging techniques provide the capacity to record the movement and behaviour of a range of animals in relation to environmental parameters^12^. Bio-loggers and transmitters now allow collection of different types of data at the individual level, including geographic location and environmental data^13,14^. The use of these devices is now commonplace, leading to an explosion in the quantity and quality of data, creating new challenges for data management, integration, and analysis, and requiring the development of new tools and approaches^15^. Scientists have thus taken advantage of the miniaturisation of electronic tags to remotely follow penguins, petrels, albatross, seals and whales at sea for more than two decades in the Southern Ocean to learn how they spend their time at sea and understand the role they play in different food webs. While lacking a species-interaction context, such data can help to identify regions utilized by multiple species of predators, which are indicative of Areas of Ecological Significance^16^, or biological hotspots, e.g.^17,18^.

Despite the considerable number of tracking studies on the distribution and habitat use patterns of upper trophic level, air breathing vertebrates in parts of the Southern Ocean based on tracking data, e.g.^19,20^, no circum-Antarctic synthesis yet exists that crosses species boundaries. This deficiency prompted the Expert Group on Birds and Marine Mammals (EG-BAMM) and the Expert Group on Antarctic Biodiversity Informatics (EGABI) of the Scientific Committee on Antarctic Research (SCAR; www.scar.org) to initiate in 2010 the Retrospective Analysis of Antarctic Tracking Data (RAATD). RAATD aims to advance our understanding of fundamental and applied questions in a data-driven way, matching research priorities already identified by the SCAR Horizon Scan^9,21^ and key questions in animal movement ecology^22^. For these reasons, we worked on the collation, validation and preparation of tracking data collected south of 45 °S. Data from over seventy contributors (Data Contacts and Citations^23^) were collated. This database includes information from seventeen predator species, 4,060 individuals and over 2.9 million at-sea locations. To exploit this unique dataset, RAATD is undertaking a multi-species assessment of habitat use for higher predators in the Southern Ocean^24^.

RAATD will provide a greater understanding of predator distributions under varying climate regimes, and provide outputs that can inform spatial management and planning decisions by management authorities such as the Commission for the Conservation of Antarctic Marine Living Resources (CCAMLR; www.ccamlr.org). Our synopsis and analysis of multi-predator tracking data will also highlight regional or seasonal data-gaps.

## Methods

### Original deployment of tracking devices

**Fig. 1:**
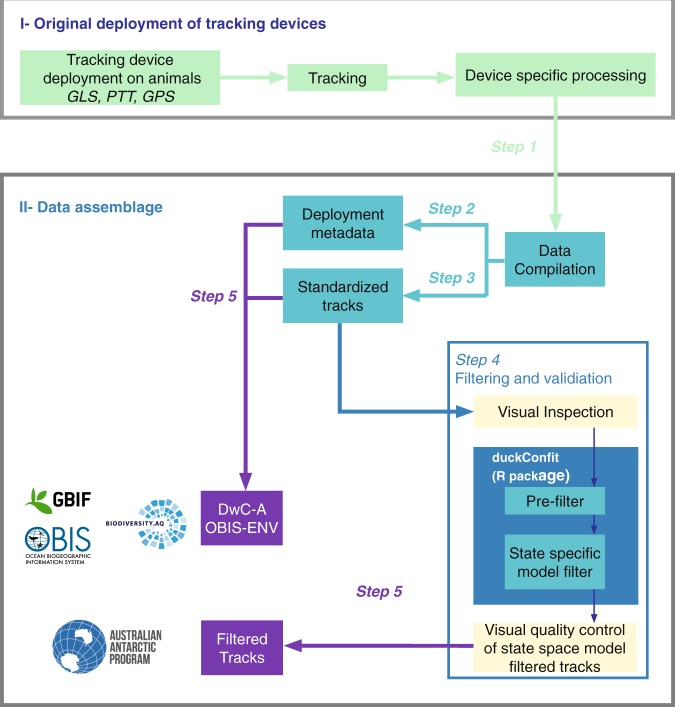
Data workflow from tracking-device deployment on animals to state-space model-filtered tracks (and associated data). Arrows and boxes correspond to the specific sections in the text. The blue box indicates the filtering and validation workflow for which R scripts are provided; purple boxes indicate publiclyavailable data files through the AADC and Darwin Core packages available through the Global Biodiversity Information Facility (GBIF) and Ocean Biogeographic Information System (OBIS).

RAATD aggregated data from three types of tracking devices (Fig. 1). In increasing order of precision these are light-level recording Global Location Sensors (GLS loggers or geolocators), satellite-relayed Platform Terminal Transmitters (PTTs), and Global Positioning System devices (GPS). Typically, GLS and GPS devices record data in internal memory, and must be physically recovered in order to download the data. PTTs transmit a carrier signal to satellites, and can deliver data remotely and in near-real time. Some modern devices now combine the capabilities of PTT and GPS (or other) devices, relaying high-quality GPS data to the end user via satellites. GLS devices, which are among the smallest, allowing for deployments on smaller predators, typically record ambient light levels throughout the day, from which coarse estimates of latitude and longitude can be calculated (to within 100–200 km) using day length and timing of local noon. Some GLS units can also record sea surface temperature, which can help refine position estimates^25^. GLS locations were estimated by RAATD data contributors using five methods^26–30^ (GLS Methods^23^) and generally corresponded to individual distribution during the non-breeding season. GPS tags make use of global navigation satellite systems and provide very high resolution (about ten meters) location fixes and time information. Some are satellite-linked, while others have smaller batteries and must be recovered (i.e. the animal carrying the tag must be recaptured) to download the archived data. PTT tags transmit signals to ARGOS satellites which transfer the received signals to a receiving station at the Collecte de Localisation Satellites (CLS) in Toulouse, France, to estimate locations based on Doppler shifts in the received signals to an accuracy of approximately 1,000 m. Processing by CLS involved a least-squares filtering method up to 2008, thereafter Kalman filters have been used^31^. Different models of GLS, PTT, and GPS devices from different manufactures have been used throughout the years, each having specific characteristics (size, operating modes, etc.) that may influence accuracy of the locations, but because device type was not always provided by the data providers, a standard correction has been applied in RAATD (see below). In summary, the “RAATD core group” (i.e. the analysing team) worked on location data converted from light-level data by the data contributors, on CLS-processed PTT location data, and on raw data directly delivered by GPS devices.

Device attachment to animals was also species-specific. When loggers are small enough, like GLS, they are mounted on leg or flipper bands/tags, while larger data-loggers and transmitters are often attached to the plumage or pelage on the back or head of the animal, a position that optimizes data communication with satellites. Modes of attachment on the back varied from using harnesses, glue or marine tape. For whales, transmitters with subcutaneous anchors were attached to the back, using poles, cross bows or air guns. Scientists limited handling time and stress as much as possible during attachment and retrieval of devices, e.g.^32–37^, following established animal handling guidelines that meet ethical reviews. However, it should be noted that the RAATD dataset contains tracking data that span almost three decades, during which time substantial progress has been made in terms of miniaturization and advances in electronic components. Any adverse effects of devices on animals are therefore likely to be less acute in recent years compared to the earlier years of tracking.

### Data assemblage

#### Step 1. data collection

Starting from 2010, RAATD compiled a catalogue of existing (both published and unpublished) tracking data by contacting international experts that held data. Data were also harvested from existing repositories, including the Australian Antarctic Data Center (https://data.aad.gov.au/), the Integrated Marine Observing System (http://imos.org.au/), PANGAEA (https://www.pangaea.de/), BirdLife International (http://www.seabirdtracking.org/), the Antarctic Biodiversity Portal (http://www.biodiversity.aq/), Ocean Biogeographic Information System (http://www.iobis.org/), and the Global Biodiversity Information Facility (http://www.gbif.org/). The data-collection phase ended in 2016.

#### Step 2. associated metadata

Where available, information on the deployment site and relevant characteristics of the animal at the time of deployment was standardized. Where age class and sex were known, this information was included in the metadata.

#### Step 3. data standardization

Location dates and times were converted to UTC (Coordinated Universal Time). Records with missing latitude or longitude values were removed, and all longitudes were transformed to lie between 180 °W and 180 °E. Data files were row-ordered by individual, with rows within an individual in their correct temporal sequence. Near-duplicate positions, defined as animal positions that occurred three seconds or less after an existing position fix from the same animal, and which had identical longitude and latitude values (for GPS devices) or longitude and latitude values that differed by less than 1^−05^ and which had the same location quality value (for PTT devices), were removed.

Entries in the age class, breeding stage, device type, location quality, scientific, common, and abbreviated name, sex, and deployment site columns were validated against controlled vocabularies. Mandatory entries (e.g., deployment date, device type, individual animal identifier) were checked for missing values. When the data contributors could not provide missing deployment dates, the first data point of the track was used as a reference point for deployment. Where animal identifiers were missing, they were created from the the tag identifier or file name.

Deployment locations were recorded by the original field team either at the individual-animal level (using e.g., a hand-held GPS device) or at the deployment-site level (i.e., one deployment location per group of animals). The latter was common for deployments at colonies, whereas the former was most common for non-colony deployments (e.g., seals and whales). Where deployment locations were not recorded by the field team, the first location estimate(s) in the tracking data were used. Deployment site names were standardized to colony names wherever possible (e.g., to the beach-on-island level).

Periods at the start or end of deployments were identified and discarded if there was evidence that location data during these periods did not represent the animals’ at-sea movement. For example, tags may have been turned on early (thereby recording locations prior to their deployment on animals) or animals may have remained at the deployment site, e.g. the breeding colony, for an extended period at the start or end of the tag deployment. Some tracks also showed a marked deterioration in the frequency and quality (for PTTs) of location estimates near the end of a track. Such locations were visually identified based on maps of each track in conjunction with plots of location distance from deployment site against time. This information is captured in the location_to_keep column appended to each species’ raw data file (1 = keep, 0 = discard).

#### Step 4. data filtering

Each track in the standardized dataset was visually inspected by the Data Editorial Group, and flagged for removal (using the *keepornot* column in the metadata file) if location estimates appeared unreasonably noisy relative to the length and extent of the track, and/or the location estimates were very irregular in time.

Next, automated quality-control checks were used to remove individual deployments that: (1) were flagged for removal (*keepornot* column in the metadata file); (2) had fewer than twenty location records; or (3) had deployments lasting less than 1 day. Additionally, individual deployments were checked to ensure that: (1) near-duplicate records in PTTs (locations occurring within 2 min of each other) were removed; (2) PTT Argos Z-class locations were reclassified as B-class locations (the least precise Argos location quality class that has an associated error variance^38^); and (3) locations implying unrealistic travel rates during the preceding time step (over 10 m s^−1^ for penguins and marine mammals and over 30 m s^−1^ for flying seabirds) were removed. Note that the definition of “duplicate locations” in the filtering context is more aggressive (less than two minutes vs less than three seconds) than that used during data standardization: for standardization, the intention was to keep the data as close to original as possible, whereas for filtering the presence of multiple positions in a short period of time (less than two minutes) has a negative effect on the filter performance.

A state-space model (SSM) was used to estimate locations at regular time intervals (one hour for GPS data; two hours for Argos data; twelve hours for GLS data) and account for measurement error in the original observations^12,38^. The data were SSM-filtered and subjected to a final quality control where tracks that failed to converge, as judged by nlminb convergence criteria^39^, were re-fitted using different initial values. If re-fitted tracks continued to fail to converge they were removed from the final filtered dataset.

For converged tracks, longitude and latitude residuals were examined for systematic trends indicative of lack of fit. Tracks that failed this inspection were removed from the final filtered dataset.

#### Step 5. data publication

RAATD established a data-sharing and publication agreement with all data providers in 2017. The standardized (trimmed) and filtered data are held in a data repository hosted at the Australian Antarctic Division (AADC) (see details below, in the ‘Standardized Data’ section). The filtered data are also according to the OBIS-ENV guidelines^40^ published in international repositories through the SCAR Antarctic Biodiversity Portal (see details below, in the ‘OBIS-ENV compliant data’ section). For this purpose, and to ensure standardized file structure, secure (meta)data storage and the facilitation of community access to the data (where appropriate), the resulting datasets have been uploaded to the biodiversity.aq IPT instance (Integrated Publishing Toolkit; www.ipt.biodiversity.aq), the accepted route for publishing data to the SCAR Antarctic Biodiversity Portal (www.biodiversity.aq). This should ensure a seamless flow to the Ocean Biogeographic Information System (OBIS) and the Global Biodiversity Information Facility (GBIF).

## Data Records

### Original data provided by contributors

The original data provided by the data contributors are not made available here. If needed, the relevant contributors should be contacted; contact details are provided in the metadata file (columns *data_contact* and *contact_email*) and in Data Contacts and Citations^23^.

### Standardized data

Standardized data are provided in files of aggregated comma-separated values (CSV). They are made available as (i) a single metadata file containing a description for each individual in the dataset and (ii) a set of seventeen CSV files, one for each species, which aggregate all of the respective individual location data (Online-only Table 1). Records in the two files can be linked by the common ‘individual_id’ field, as each animal in the study has a unique identifier. The data and metadata are available to the public through the Australian Antarctic Data Centre: standardized data^41^; state-space model-processed (filtered) data^42^.

### OBIS-ENV compliant data

The standardised data will also be provided as a set of Darwin Core Archives using the Darwincore Event core (Fig. 1) in compliance with the OBIS-ENV-DATA format^40^. All field definitions (Darwin Core Terms) are available on the Darwin Core website (at: http://rs.tdwg.org/dwc/terms/index.htm#occurrenceindex). The Darwin Core aims to share data about taxa in a simple structured way. It includes a glossary of terms and is primarily based on their occurrence in nature as documented by observations, specimens, samples, and related information. Documents describing how these terms are managed, how the set of terms can be extended for new purposes, and how the terms can be used can be found on the website.

The OBIS-ENV compliant data data are made publicly available through the Antarctic Biodiversity Portal Integrated Publishing toolkit (http://ipt.biodiversity.aq/resource?r=raatd_scar_trackingdata). The Antarctic Biodiversity Portal acts as the Antarctic thematic node for the Ocean Biogeographic Information System (OBIS, Ant-OBIS) and the Global Biodiversity Information Facility (GBIF, AntaBIF).

### Geographic coverage

**Fig. 2:**
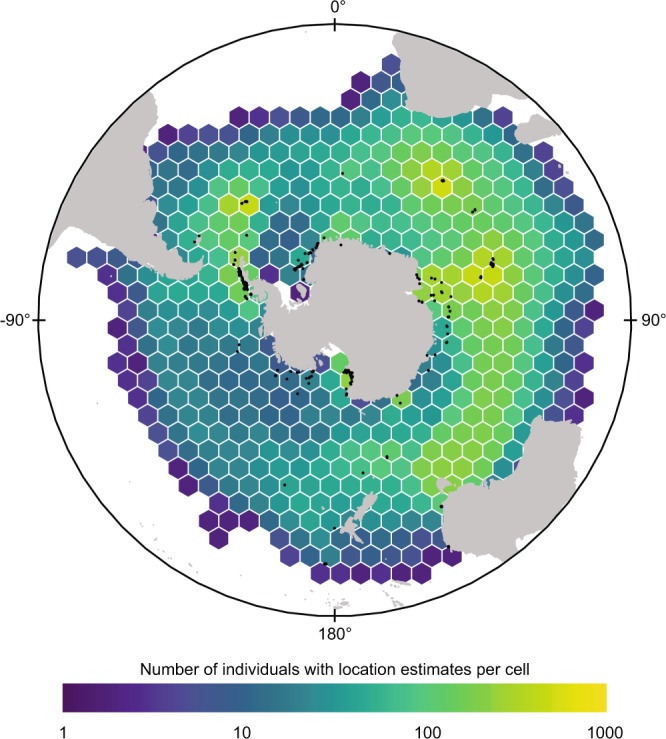
Spatial distribution of the number of individuals tracked per 25,000 km^2^ hexagonal grid cell throughout the domain of the dataset. Deployment locations are shown with black points. The map is a Lambert Azimuthal equal area projection, showing the area 90° S to 20° S.

All species considered in this dataset have circumpolar Antarctic distributions (Fig. 2; species-specific distributions are given in Supplementary Fig. S1) with a longitudinal range spanning 180 °W to 180 °E. The species breed either on the coast of the Antarctic continent or on the sub-Antarctic islands to the north (see Supplementary Table S1 for a list of the main study sites). Species with geographically limited distributions (such as chinstrap penguins *Pygoscelis antarcticus*) were not included; instead we concentrated on species whose distribution covers a large portion of the Southern Ocean. In addition, a number of deployments in the Antarctic (crabeater seals *Lobodon carcinophaga* and Weddell seals *Leptonychotes weddellii*) were conducted in the pack ice at un-named locations. Similarly, humpback whales *Megaptera novaeangliae* were instrumented at sea either off the coast of the Antarctic Peninsula, off Australia or off New Zealand.

### Taxonomic coverage

**Fig. 3:**
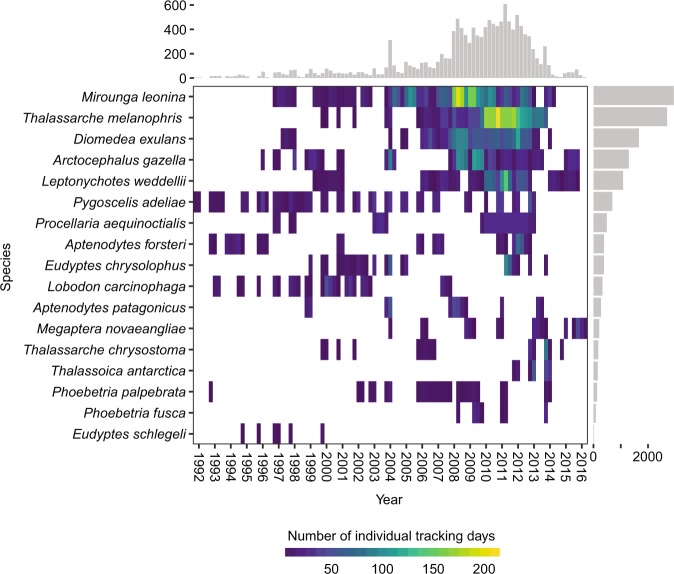
Number of individual tracking days (number of individuals with at least one SSM location estimate on a given day) per species and quarter.

Seventeen species of meso- and top predators were selected for analyses (Table 1 and Fig. 3), five marine mammals (one baleen whale, one otariid and three phocid seals) and twelve seabirds (five penguins, five albatrosses, and two petrels). These species cover a diverse range of ecological niches and life-history traits and include dietary specialists (e.g., crabeater seals), deep divers (e.g., elephant seal *Mirounga leonina* and emperor penguin *Aptenodytes forsteri*), wide ranging, highly migratory species (e.g., wandering albatross *Diomedea exulans*), nearshore foragers (e.g., Adélie penguin *Pygoscelis adeliae*) and capital (e.g., Weddell seal) versus income (e.g., Antarctic fur seal *Arctocephalus gazella*) breeders. In total, 4,060 individuals were included in the standardized dataset before quality control (some individuals may have been counted more than once in this total, as repeat deployments on the same individuals are not taken into account in this summation), with 1,482 marine mammals and 2,578 seabirds, providing 2,964,245 location fixes before filtering (Table 1). After filtering and quality control processes, the total number of individuals used was 2,823, providing 2,328,772 location fixes (a 21% decrease in the number of location fixes) (Table 1).

**Table 1:** Count of the number of individuals and location fixes by species included in the RAATD project initially (standardized data) and following the cleaning and filtering processes (filtered data).

Species			Standardized data		Filtered data		
	Common name (abbreviation)	Scientific name	N ind.	N fixes	N ind.	N fixes	File name
Total Aves			2,578	1,277,595	1,768	716,057	
Aves	Adélie penguin (ADPE)	*Pygoscelis adeliae*	820	249,089	520	90,202	RAATD2017_ADPE
	Emperor penguin (EMPE)	*Aptenodytes forsteri*	129	74,672	93	104,722	RAATD2017_EMPE
	King penguin (KIPE)	*Aptenodytes patagonicus*	117	81,905	109	57,562	RAATD2017_KIPE
	Macaroni penguin (MAPE)	*Eudyptes chrysolophus*	505	160,766	182	39,090	RAATD2017_MAPE
	Royal penguin* (ROPE)	*Eudyptes schlegeli*	20	4,566	12	3,341	RAATD2017_ROPE
	Antarctic petrel (ANPE)	*Thalassoica antarctica*	127	139,603	121	27,957	RAATD2017_ANPE
	White-chinned petrel (WHCP)	*Procellaria aequinoctialis*	67	37,606	59	36,875	RAATD2017_WHCP
	Wandering albatross (WAAL)	*Diomedea exulans*	276	189,120	253	120,568	RAATD2017_WAAL
	Black-browed albatross (BBAL)	*Thalassarche melanophris*	328	144,773	244	161,148	RAATD2017_BBAL
	Grey-headed albatross (GHAL)	*Thalassarche chrysostoma*	112	159,547	107	29,501	RAATD2017_GHAL
	Sooty albatross (DMSA)	*Phoebetria fusca*	35	14,717	33	23,119	RAATD2017_DMSA
	Light-mantled albatross (LMSA)	*Phoebetria palpebrata*	42	21,231	35	21,972	RAATD2017_LMSA
Total Mammalia			1,482	1,686,650	1,055	1,612,715	
Mammalia	Antarctic fur seal (ANFS)	*Arctocephalus gazella*	555	258,443	313	209,472	RAATD2017_ANFS
	Crabeater seal (CRAS)	*Lobodon carcinophaga*	105	83,212	90	89,370	RAATD2017_CRAS
	Southern elephant seal (SOES)	*Mirounga leonina*	570	1,013,075	437	913,760	RAATD2017_SOES
	Weddell seal (WESE)	*Leptonychotes weddellii*	176	231,967	157	342,563	RAATD2017_WESE
	Humpback whale (HUWH)	*Megaptera novaeangliae*	76	99,953	58	57,550	RAATD2017_HUWH
Grand total			4060	2,964,245	2,823	2,328,772	

Note, in some cases (e.g., emperor penguin) the number of fixes of the filtered data is greater than the original number of fixes in the raw data due to a high prevalence of tag duty-cycling (tags working non-continuously, e.g., recording 12 h during the day and being turned off for 12 h at night) or due to periods when no location fixes were recorded but which were interpolated by the state-space model.

*Royal penguins have a limited geographic distribution, but they can be considered ecologically equivalent to Macaroni penguins where they occur, and these two species will be considered together in RAATD’s further analyses.

### Temporal coverage

The data are not distributed evenly in time (Fig. 3). While the time frame ranges from 1991 to 2016, most data were collected during the period 2007 to 2014. This is a reflection of increased research effort in the Southern Ocean and advances in technology since the early 1990s, and also the timeline of the RAATD project which stopped actively seeking new data inputs in 2016. Further, some data providers primarily contributed older datasets that were already published or soon to be published, rather than unpublished data.

The lack of even coverage in terms of taxa, space and time is a function of several factors. First, some deployments were mostly conducted during the breeding season when species like Adélie penguins make relatively short duration (2–14 days) and local (10–200 km) foraging trips, compared with post-moulting southern elephant seals that make distant (several thousands of km) and longer duration foraging trips (many weeks). Second, the coverage reflects the research effort related to funding and logistics and, third, the availability of species that lend themselves to instrumentation (e.g., central place foragers). For instance, crabeater seals are very abundant but because they inhabit pack ice they are logistically very difficult to capture for tracking studies. In the case of humpback whales, long-term attachments of tracking equipment are relatively difficult to attain so there are less data available. The technology to track the smaller flying bird species is also comparatively new, relying until recently upon small archival light loggers (see above), so there have been relatively few studies of these species to date.

## Technical Validation

The standardized data were subjected to a range of quality checks before undertaking further processing (Fig. 1). These included:

- Counts of unique deployment positions and longitude/latitude variability were calculated for each dataset, and used as a check for errors in deployment position.
- The distance from the recorded deployment position to the first few track points was calculated, and any distances greater than 10 km were flagged for manual inspection and verification. Similarly, the deployment date was compared to the date of the first point of the track, and differences were flagged for manual verification.
- Various cross-checks were conducted to identify other data errors or discrepancies, including checking for multiple device identifiers associated with a single individual animal identifier, checking for identical individual identifiers on different species or in different datasets, checking that redeployed devices (i.e. the same device deployed on multiple individuals) did not have temporal overlap, and checking for data missing from the 29^th^ of February of leap years (perhaps indicating data that had been discarded by accident).

A number of additional quality-control checks were implemented prior to the data filtering; these are described in the Methods (Steps 3–4). State-space models (SSM) are now the standard approach for dealing with observation errors in electronic tagging location data^38,43^. The SSM filtering protocol that was applied to all the data provided essential quality control and validation. It was a variation of what was used by Jonsen *et al*.^38^, which was implemented in the statistical computing language R^44^ via the Template Model Builder package (TMB package)^45^. The TMB package provides extremely fast and stable maximum likelihood estimation, via autodifferentiation and the Laplace approximation, for non-Gaussian and nonlinear SSM’s^46^. This was essential for filtering the large amount of tracking data compiled herein.

The SSM filtering accounted for observation errors in the tracking data and, unlike the raw track data, provided location estimates and standard errors at regular time intervals along estimated tracks^38,47^. These location (error-filtered and time-regularised) outputs are essential for determining species’ habitat preferences from tracking data (see Usage Notes) and other types of ecological inferences. We encourage users of these filtered outputs to evaluate the level of uncertainty in the estimated locations for their ecological inferences, as our methods for filtering are specific for our purposes. Example filtering code is provided so that users can reproduce our filtered data from the raw data or produce a new set of filtered data using, for example, different time steps (https://github.com/SCAR/RAATD).

Following SSM filtering, estimated tracks were evaluated for goodness of fit by examination of (1) maps of estimated and observed locations and (2) residual plots of latitude and longitude. Tracks associated with obviously poor fits to the data, unrealistic estimated movements and frequent extended periods without observations (relative to the step length duration) were discarded from the final output dataset. This examination was conducted independently by three people. Estimated tracks were discarded when at least two examiners were in agreement to discard. In cases where the optimisation algorithm (nlminb in R) failed to converge to a global minimum, up to ten attempts with different initial values were made in an effort to obtain convergence. Tracks for which convergence could not be obtained were discarded from the final output dataset. Combined, these quality control and validation procedures accounted for a 30% reduction in the number of individual tracks retained in the filtered data compared with the standardized data (Table 1).

The SSM-filtered data are affected by several caveats. First, the standardised GPS, PTT and GLS data were filtered using time steps of 1-, 2- and 12 h, respectively. These time steps were chosen as they are generally appropriate relative to the typical sampling frequencies of the three tag types. In some cases, these time steps did not match well with the sampling frequency of particular tags. For example, GPS tags deployed on some birds had far higher sampling frequencies and a 1-h time step may be too coarse in these cases. Second, for GLS data the time period around the equinoxes (approximately four weeks, each) yields suspect latitude estimates. The SSM filter does not fully account for this uncertainty. Third, for animals carrying tags programmed to turn off when hauled out on land or ice the SSM-estimated locations imply movement looping beyond and back to these haul-out sites when the tags are off. These estimates are clearly spurious. Fourth, tag-sampling frequency often declines toward the end of long deployments. Despite some influence on SSM-estimated locations near the end of these deployments, these data were retained in RAATD.

## Usage Notes

Thanks to an unprecedented sharing effort from the SCAR EG-BAMM community, a benchmark dataset has been assembled that fills important gaps in spatial occurrence of various species for areas of the world that are traditionally data-poor. The dataset compiled for RAATD is used in the analytical project described in the Background section to determine Areas of Ecological Significance for the 17 species of predators considered in the dataset. To this end, a habitat selectivity procedure is one possible modelling method, aiming to identify the particular environmental conditions that are favoured by the animals, relative to the range of conditions that are available. This first requires estimation of the geographic space available to a given animal, which can be assessed using various methods, e.g.^48^. This region of geographic space has an associated range of environmental conditions over the period in which animals were tracked. Regression modelling can then be used to identify the environmental covariates that discriminate areas that are preferentially utilized. For particular analyses, tracking data may need to be subdivided, for example by breeding stage, depending on whether or not the animals’ interactions with the environment differ by breeding stage. The individual habitat preference models can then be combined to provide a multi-species view of important regions of habitat including their underlying environmental processes, e.g.^17,18^. Following this analysis and production of a scientific article, the dataset will be available for re-use to help address emerging research questions or pressing conservation issues.

The SCAR EG-BAMM is pleased to make this dataset openly available for the Antarctic and broader scientific communities. It is organized and curated using the best principles and practices of recent biodiversity informatics practices^49^. In this framework, the final version of the dataset is fully compliant with Darwin Core body of standards and can be downloaded through the Global Biodiversity Information Facility (GBIF) and Ocean Biogeographic Information System (OBIS) data portals.

In regard to the use of the dataset, the RAATD consortium promotes the CC-BY (Creative Commons Attribution License), this being the standard practice for citing GBIF-mediated data, believing that it reflects an established norm that the communities we serve use to cite original work. Users are expected to comply with the guidelines of the SCAR/SCADM Data Policy: https://www.scar.org/scar-library/reports-and-bulletins/scar-reports/2717-scar-report-39/file/ and to recognize the valuable contributions of data providers (generally scientists who collect, synthesise, model, or prepare analysed data) and to facilitate repeatability of research results. Users of SCAR data should communicate with and formally acknowledge data authors (contributors) and sources, refer to Data Contacts and Citations^23^, for specific citations. Where possible, this acknowledgment should take the form of a citation, such as when citing a book or journal article.

### Supplementary information

Supplementary Table S1

Supplementary Figure S1

## Data Availability

The code for (i) trimming the raw tracks and (ii) the state space filtering have been made available on the SCAR github page (https://github.com/SCAR/RAATD). Additional information is provided in the Technical Validation section below.

## Online-only Table

**Online-only Table 1:** Description of fields (column names) in the metadata and data files.

Column name	Description
*Metadata file*	
dataset_identifier	The unique identifier of the source dataset.
file_name	Name of the raw data file containing tracking data provided by data contributors.
individual_id	Unique individual ID for each track.
keepornot	Whether the individual track is retained (Keep) or discarded (Discard) in the final SSM output (see “Data Assemblage - Step 3. Data trimming“).
scientific_name	Scientific name of the species tracked.
common_name	English common name of the species tracked.
abbreviated_name	Four-letter abbreviation identifying the species tracked (see Table 1).
sex	Sex of the tracked individual (F = female, M = male, U = unknown).
age_class	Age class of the tracked individual (Adult, Fledgling, Subadult, Yearling, Underyearling, Unknown).
device_id	ID of the tracking device.
device_type	Type of tracking device used (GLS = Global Location Sensor, GPS = Global Positioning System; PTT = Platform Terminal Transmitter).
deployment_site	Name of deployment site.
deployment_year	Four-digit year of device deployment.
deployment_month	Ordinal month of device deployment (1–12).
deployment_day	Integer day of the month of device deployment (1–31).
deployment_time	Time of day (Coordinated Universal Time, UTC) of device deployment (hours:minutes).
deployment_decimal_longitude	Longitude of deployment site (decimal degrees; −180 to 180).
deployment_decimal_latitude	Latitude of deployment site (decimal degrees).
data_contact	Name of the data contact(s).
contact_email	E-mail address of the data contact(s).
comments	Comments.
***Standardized data files***	
individual_id	Unique individual ID for each track.
abbreviated_name	Four-letter abbreviation identifying the species tracked (see Table 1).
breeding_stage	Breeding stage/status of the individual tracked.
year	Four-digit year of the location record.
month	Ordinal month of the location record (1–12).
day	Integer day of the month of the location record (1–31).
time	Time of day of the location record (hh:mm:ss), expressed in Coordinated Universal Time (UTC).
decimal_latitude	Latitude of location record (decimal degrees).
decimal_longitude	Longitude of location record (decimal degrees; −180 to 180).
location_quality	If tracking device (device_type) is PTT, the Argos location quality of the location record.
location_to_keep	Whether the location record is used (1) or ignored (0) when fitting the state-space model.
***Filtered data files***	
individual_id	Unique individual ID for each track.
abbreviated_name	Four-letter abbreviation identifying the species tracked (see Table 1).
breeding_stage	Breeding stage/status of the individual tracked.
date	Date and time of the location estimate, expressed in Coordinated Universal Time (UTC).
decimal_longitude	Longitude of location estimate (decimal degrees; −180 to 180).
decimal_latitude	Latitude of the location estimate (decimal degrees).
longitude_se	Standard error of the longitude estimate (degrees)
latitude_se	Standard error of the latitude estimate (degrees)

The metadata file contains single comma-separated values (CSV) for each record (row) per tracked individual, and twenty-one columns, with names indicated in a header row. The data files are a set of seventeen CSV files (one per species) containing one record (row) per location for all individuals of that species, with eleven columns named in a header row. The metadata records can be linked to records in the tracking data files by the *individual_id* column.
