# Supplementary material for: The retrospective analysis of Antarctic tracking data project
Source: Sci Data. 2020 Mar 18;7:94. doi: 10.1038/s41597-020-0406-x (PMC7080749; doi:10.1038/s41597-020-0406-x)
Supplement: Supplementary file 1 — Supplementary Table S1 [file 41597_2020_406_MOESM1_ESM.docx]

**Supplementary Table S1**: Names and coordinates of the major study sites in the Southern Ocean and on the Antarctic Continent where tracking devices were deployed on the selected species (indicated by their 4-letter codes in the last column).

| **Sector** | **Location name** | **Location coordinates** | **Species** |
| --- | --- | --- | --- |
| Indian Ocean | Prince Edward Islands | 46° 46′ S, 37° 51′ E | ANFS, DMSA, GHAL, KIPE, LMSA, MAPE, SOES, WAAL |
|  | Crozet Islands | 46° 25′ S, 51° 59′ E | KIPE, WAAL, WHCP |
|  | Kerguelen Islands | 49° 15′ S, 69° 10′ E | ANFS, BBAL, SOES, WAAL |
|  | Heard Island | 53° 6′ S, 73° 31′ E | ANFS, BBAL, KIPE, LMSA, MAPE |
| South Atlantic Ocean | Falkland (Islas Malvinas) | 51° 41′ S, 59° 10′ W | KIPE, SOES |
|  | Signy Island | 60° 43′ S, 45° 36′ W | ADPE |
|  | South Georgia | 54° 15′ S, 36° 45′ W | ANFS, BBAL, KIPE MAPE, SOES, WAAL, WHCP |
|  | Bouvet Island | 54° 25′ S, 3° 22′ E | ANFS, SOES |
| Antarctic Peninsula | South Shetland Islands (incl. Elephant Island) | 62° 0′ S, 58° 0′ W | ADPE, ANFS, SOES, WESE |
|  | Sea ice or at sea |  | CRAS, HUWH, WESE |
| South Pacific Ocean | Macquarie Island | 54° 30′ S, 158° 57′ E | ANFS, BBAL, GHAL, KIPE, LMSA, ROPE, SOES, WAAL |
|  | Campbell Island | 52° 32′ S, 169° 8′ E | GHAL |
|  | At sea off Raoul Island | 29° 15′ S, 177° 59′ W | HUWH |
|  | At sea off the Australian east coast |  | HUWH |
| Weddell Sea | Filchner Trough | 77° 0′ S, 36° 0′ W | WESE |
|  | Drescher Inlet | 72° 50′ S, 19° 2′ W | CRAS, WESE |
|  | Sea ice |  | CRAS, WESE |
| Queen Maud Land | Svarthamaren | 71° 54′ S, 5° 10′ E | ANPE |
|  | Hukuro Cove | 69° 12′ S, 39° 37′ E | ADPE |
| Mac.Robertson Land | Taylor Glacier | 67° 27′ S, 60° 51′ E | EMPE |
|  | Ufs Island | 67° 28′ S, 61° 8′ E | ADPE |
|  | Béchervaise Island | 67° 35′ S, 62° 49′ E | ADPE |
|  | Auster Rookery | 67° 24′ S, 63° 57′ E | EMPE |
|  | Scullin Monolith | 67° 47′ S, 66° 42′ E | ADPE |
| Princess Elizabeth Land | Amanda Bay | 69° 15′ S, 76° 51′ E | EMPE |
|  | Magnetic Island | 68° 33′ S, 77° 55′ E | ADPE |
|  | Vestfold Hills | 68° 33′ S, 78° 15′ E | SOES, WESE |
| Wilkes Land | Shirley Island | 66° 17′ S, 110° 29′ E | ADPE |
| East Antarctica | Sea ice |  | CRAS |
| Terre Adélie | Pétrel Island, Terre Adélie | 66° 40′ S, 140° 1′ E | ADPE, EMPE, WESE |
| Ross Sea | McMurdo Sound | 77° 30′ S, 165° 0′ E | WESE |
|  | Edmonson Point | 74° 20′ S, 165° 8′ E | ADPE |
|  | Ross Island | 77° 30′ S, 168° 0′ E | ADPE, EMPE |
|  | Coulman Island | 73° 29′ S, 169° 45′ E | EMPE |
|  | Sea ice |  | WESE |
