# Supplementary material for: The retrospective analysis of Antarctic tracking data project
Source: Sci Data. 2020 Mar 18;7:94. doi: 10.1038/s41597-020-0406-x (PMC7080749; doi:10.1038/s41597-020-0406-x)
Supplement: Supplementary file 2 — Supplementary Figure S1 [file 41597_2020_406_MOESM2_ESM.pdf]

Adélie penguin

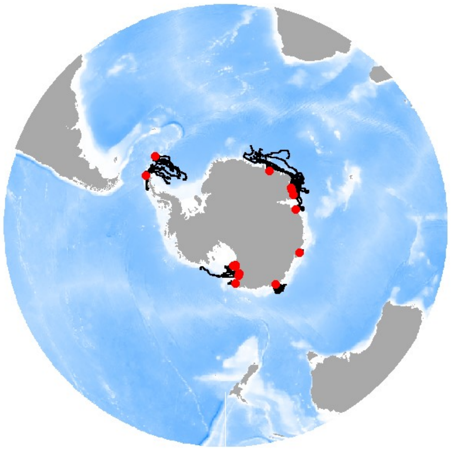

Antarctic fur seal

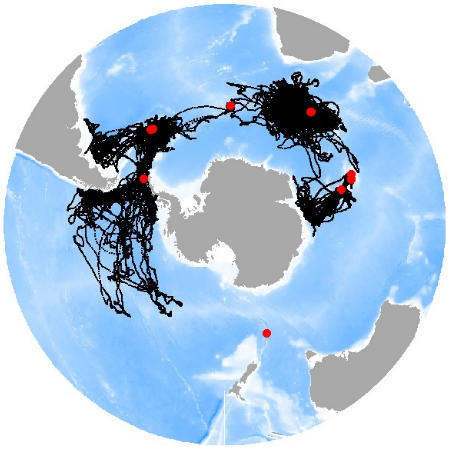

Antarctic petrel

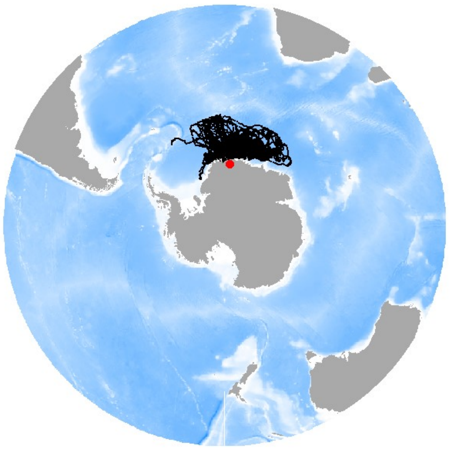

Black-browed albatross

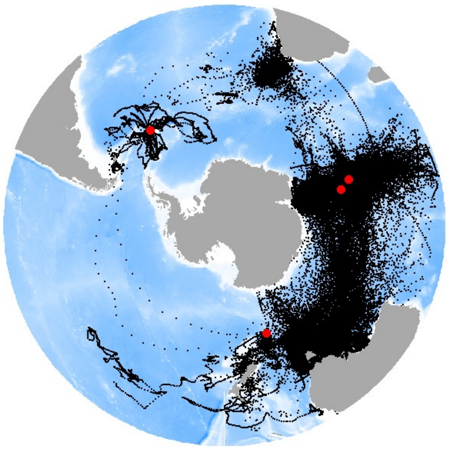

Crabeater seal

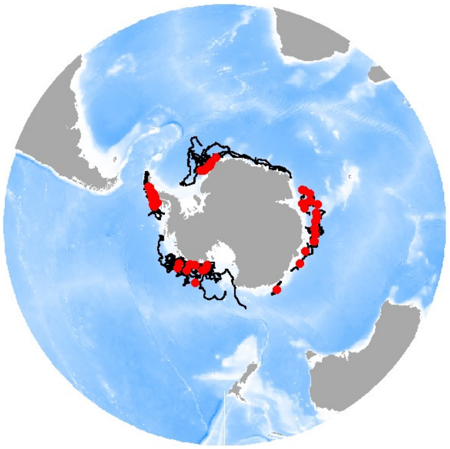

Emperor penguin

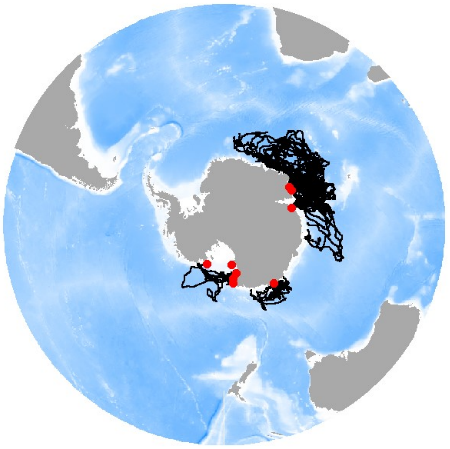

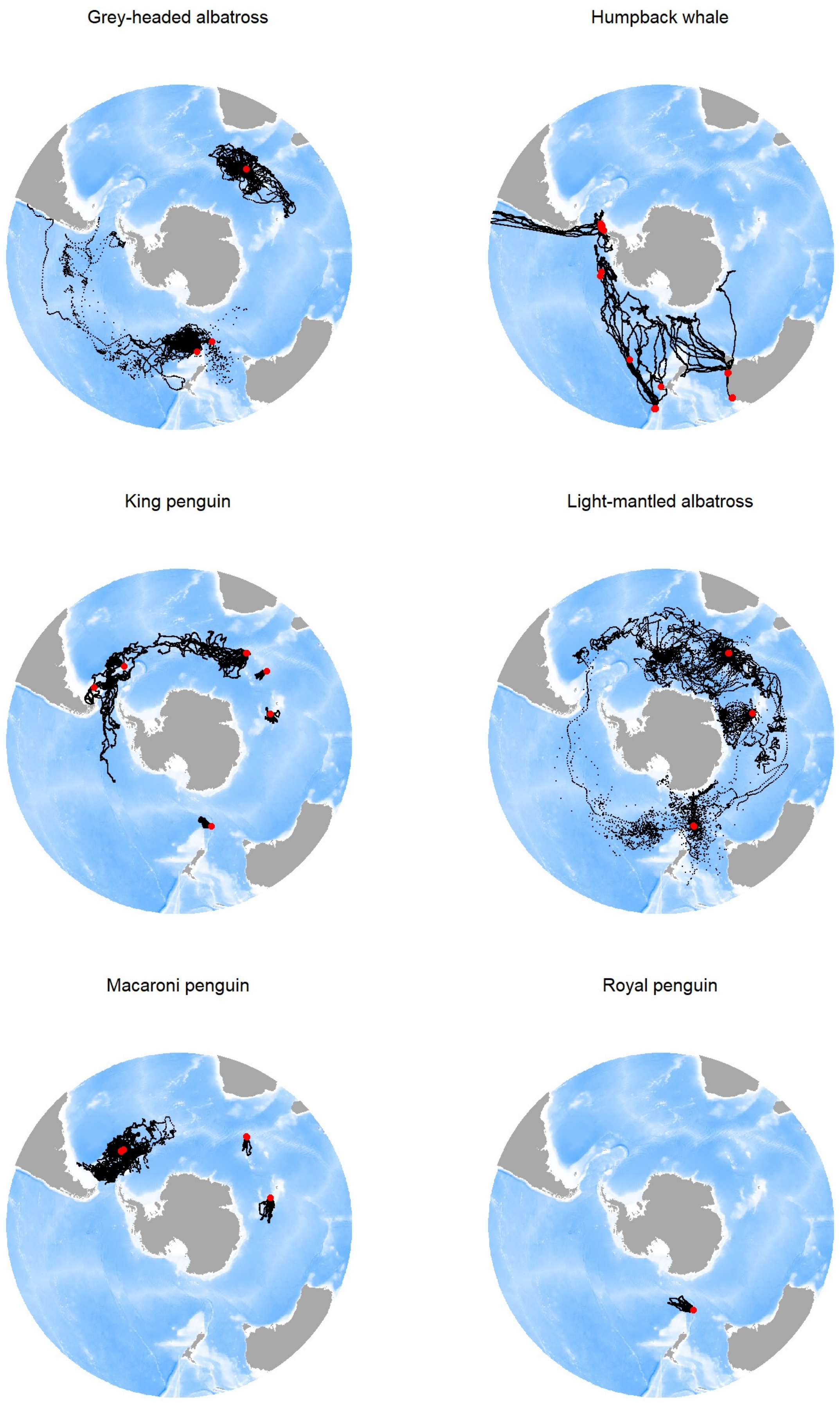
Grey-headed albatross
Humpback whale
King penguin
Light-mantled albatross
Macaroni penguin
Royal penguin

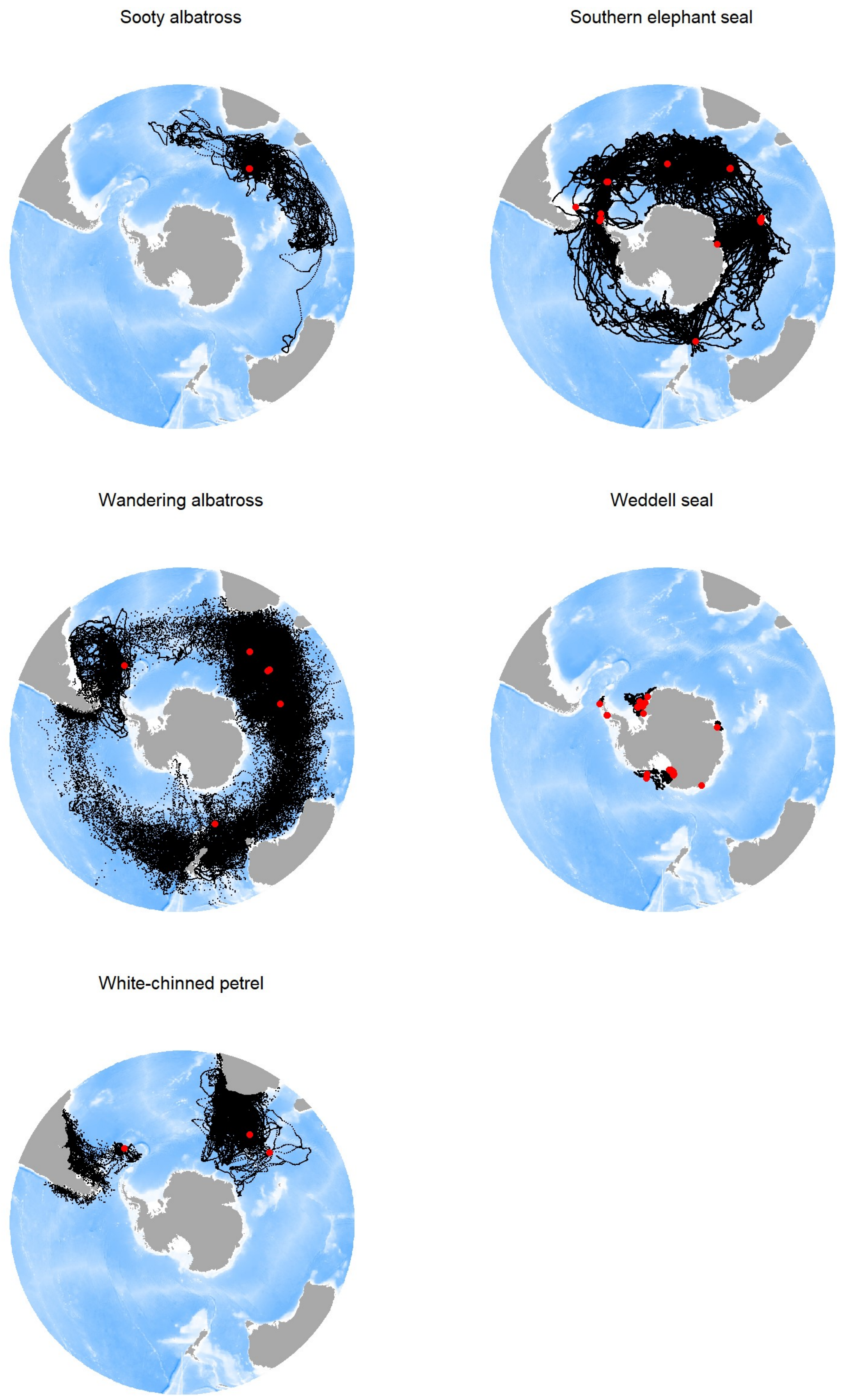

Supplementary Figure S1: Filtered location data (black) and tag deployment locations (red) for each species. Maps are Lambert Azimuthal projections extending from 90° S to 20° S.
